# A proteome analysis of freezing tolerance in red clover (*Trifolium pratense* L.)

**DOI:** 10.1186/s12870-016-0751-2

**Published:** 2016-03-10

**Authors:** Annick Bertrand, Marie Bipfubusa, Yves Castonguay, Solen Rocher, Aleksandra Szopinska-Morawska, Yousef Papadopoulos, Jenny Renaut

**Affiliations:** Agriculture and Agri-Food Canada, Québec City, Canada; Luxembourg Institute of Science and Technology, Belvaux, Luxembourg; Agriculture and Agri-Food Canada, Kentville, Canada

**Keywords:** Red clover, Cold acclimation, Proteomic analysis, Recurrent selection, Freezing tolerance, Plant abiotic stress

## Abstract

**Background:**

Improvement of freezing tolerance of red clover (*Trifolium pratense* L.) would increase its persistence under cold climate. In this study, we assessed the freezing tolerance and compared the proteome composition of non-acclimated and cold-acclimated plants of two initial cultivars of red clover: Endure (E-TF0) and Christie (C-TF0) and of populations issued from these cultivars after three (TF3) and four (TF4) cycles of phenotypic recurrent selection for superior freezing tolerance. Through this approach, we wanted to identify proteins that are associated with the improvement of freezing tolerance in red clover.

**Results:**

Freezing tolerance expressed as the lethal temperature for 50 % of the plants (LT_50_) increased markedly from approximately −2 to −16 °C following cold acclimation. Recurrent selection allowed a significant 2 to 3 °C increase of the LT_50_ after four cycles of recurrent selection. Two-dimensional difference gel electrophoresis (2D-DIGE) was used to study variations in protein abundance. Principal component analysis based on 2D-DIGE revealed that the largest variability in the protein data set was attributable to the cold acclimation treatment and that the two genetic backgrounds had differential protein composition in the acclimated state only. Vegetative storage proteins (VSP), which are essential nitrogen reserves for plant regrowth, and dehydrins were among the most striking changes in proteome composition of cold acclimated crowns of red clovers. A subset of proteins varied in abundance in response to selection including a dehydrin that increased in abundance in TF3 and TF4 populations as compared to TF0 in the Endure background.

**Conclusion:**

Recurrent selection performed indoor is an effective approach to improve the freezing tolerance of red clover. Significant improvement of freezing tolerance by recurrent selection was associated with differential accumulation of a small number of cold-regulated proteins that may play an important role in the determination of the level of freezing tolerance.

**Electronic supplementary material:**

The online version of this article (doi:10.1186/s12870-016-0751-2) contains supplementary material, which is available to authorized users.

## Background

The perennial growth habit of forage legumes combined with their capacity to fix atmospheric N_2_ symbiotically markedly contributes to long-term sustainability of agriculture through reduced reliance on fossil fuel consumption, improved soil fertility and structure, and by providing a renewable source of dietary protein for ruminants. Red clover (*Trifolium pratense* L.) is a leading forage legume in Canada, United States and northern and eastern Europe. Compared to alfalfa (*Medicago sativa* L.), another major legume species, red clover is characterized by a rapid spring establishment and its superior performance on acid and wet soils [1]. Red clover is however considered as a short-lived perennial in part because of its poor winter hardiness.

Freezing tolerance is one of the most important factors that determine the survival of red clover to severe winter conditions. Field selection for freezing tolerance is a long and costly process due to the unpredictability of test winters. In that context, we developed a recurrent selection scheme entirely performed indoor in growth chambers and walk-in freezers to identify genotypes with superior tolerance to freezing (TF) [2]. Recurrent selection is a cyclical breeding method that progressively increases the frequency and the optimal assortment of genes affecting quantitative traits [3]. We previously documented the efficiency of this selection method to improve freezing tolerance of alfalfa [4] and ryegrass (*Lolium perenne* L.) [5]. In the current study, the freezing tolerance of two red clover cultivars, Endure (E-TF0) and Christie (C-TF0), was compared to that of populations E-TF3 and C-TF3 and of populations E-TF4 and C-TF4 obtained respectively after three and four cycles of phenotypic recurrent selection in these cultivars.

To increase their freezing tolerance, plants have to go through the process of cold acclimation which is induced by environmental cues such as lower temperature and shorter photoperiod. This process, characterized by a progressive acquisition of freezing tolerance, involves numerous molecular changes that have been extensively reviewed [6, 7]. We previously documented higher levels of cold-induced fructans and amino acids such as glutamine and proline in raygrass [5] and superior accumulation of oligosaccharides of the raffinose family (RFO) in alfalfa [8] in response to recurrent selection for superior freezing tolerance. Furthermore, in alfalfa, the expression of cold-induced genes was more strongly up-regulated during fall acclimation in advanced cycles of selection [8]. It has been documented in several species [9, 10] including alfalfa [11], that proteome analysis further increases the understanding of the molecular bases of cold acclimation through the identification of proteins involved in the regulatory process, enzymatic activities and structural changes that take place during the acquisition of freezing tolerance.

In the current study, we assessed the impact of successive cycles of recurrent selection for freezing tolerance performed in two genetic backgrounds on the capacity of red clover to withstand exposure to low sub-freezing temperatures. We also documented changes that take place in the proteome during cold acclimation in the initial backgrounds and in advanced cycles of recurrent selection. Our objectives were to: (1) confirm the efficiency of recurrent selection to improve freezing tolerance in red clover, and (2) analyse changes in protein composition in cold acclimated plants, and (3) identify proteins that are associated with the acquisition of superior freezing tolerance in populations recurrently-selected in two genetic backgrounds.

## Results

### Freezing tolerance and regrowth biomass

**Fig. 1:**
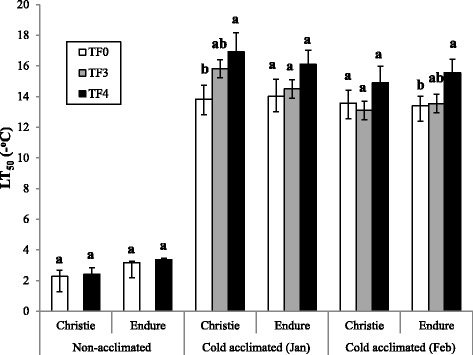
Freezing tolerance of recurrently selected populations of red clover cultivars in response to cold acclimation. Freezing tolerance is expressed as the lethal temperature for 50 % of plants (LT_50_) of the original background (TF0) and populations obtained after three (TF3) and four (TF4) cycles of recurrent selection within two cultivars of red clover: Christie and Endure. Measurements were taken with non-acclimated plants in October and cold-acclimated plants in January and February. Only plants of the initial backgrounds (TF0) and populations TF4 were assessed in the non-acclimated state. Different letters represent significant differences at *P* < 0.05 and error bars represent Standard Errors

**Fig. 2:**
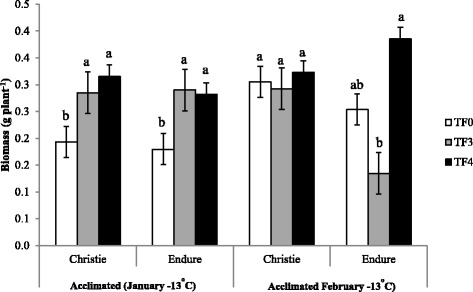
Regrowth biomass of two red clover cultivars under recurrent selection after exposure to freezing temperature. Regrowth dry matter (g plant^−1^) was measured three weeks after exposure to a −13 °C freezing stress in the initial genetic backgrounds (TF0) and populations obtained after three (TF3) and four (TF4) cycles of recurrent selection for superior freezing tolerance. Plants were previously acclimated in an unheated greenhouse during fall and winter 2010-2011. Each value is the averaged regrowth of 200 plants. Different letters represent significant differences at *P* < 0.05 and error bars represent Standard Errors

The freezing tolerance of two initial genetic backgrounds, cultivars Endure (E-TF0) and Christie (C-TF0), and of populations issued from three cycles (E-TF3 and C-TF3) and four cycles (E-TF4 and C-TF4) of recurrent selection within these two cultivars, was assessed once before acclimation in October and twice after acclimation to natural fall and winter conditions in an unheated greenhouse, in January and February (Fig. 1). The unheated greenhouse was continuously ventilated to keep the environmental conditions similar to field conditions [4]. Freezing tolerance expressed as lethal temperature for 50 % of the plants (LT_50_) was low, between −2.0 °C and −3.0 °C, when assessed with non-acclimated plants in October. It increased markedly up to −17 °C in fully acclimated plants tested in January. At that sampling date the LT_50_s increased in response to selection in both genetic backgrounds from −13.8 °C in C-TF0 to −16.9 °C in C-TF4 and from −14.0 °C in E-TF0 to −16.1 °C in E-TF4. In Christie the LT_50_ was significantly higher for C-TF4 than for C-TF0 in January while in Endure the LT_50_ was significantly higher for E-TF4 than for E-TF0 in February (Fig. 1). Measurements of regrowth biomass of acclimated plants in January confirmed the positive impact of recurrent selection on the increased capacity of plants of both genetic backgrounds to survive exposure to a low freezing temperature of −13.0 °C, with a larger regrowth biomass for TF3 and TF4 that for TF0 in both genetic backgrounds (Fig. 2). In February, the regrowth biomass did not differ between C-TF0, C-TF3 and C-TF4 but differed significantly between E-TF3 and E-TF4 (Fig. 2).

### Proteomic analyses

Difference gel electrophoresis (DIGE) based on fluorochrome labelling and separation by two-dimensional (2D) electrophoresis was used to assess variation in the abundance of specific proteins in response to cold acclimation and selection for superior freezing tolerance. More than 1600 protein spots were detected on 2D gels including 627 spots that were present in all combinations of acclimation treatments and populations (Table 1, Additional file 1: Table S2 and Additional file 2: Table S3). Among this subset, 408 spots (65.2 %) had significant homologies with sequences in databases, 148 (23.3 %) were unidentified and 71 (11.5 %) contained peptides matching to two or more proteins. Several spots sampled at different positions had homologies to the same protein in databases.

**Table 1:** Number of proteins that significantly varied in abundance in response to cold acclimation

	Cultivar			
Number of protein spots	Christie		Endure	
	Increased	Decreased	Increased	Decreased
Total protein spots (627)	273	118	287	120
Identified proteins (408)	200	77	208	71
Non-identified proteins (148)	41	31	53	39
Mixed proteins (71)	32	10	27	10

### Cold-induced changes in protein composition

In both genetic backgrounds, about 63 % of the proteome defined by 627 spots present on all gels significantly varied (*P* < 0.01) in abundance in response to cold acclimation in at least one population (Table 1, Additional file 1: Table S2 and Additional file 2: Table S3). On that basis, 274 proteins increased while 118 proteins decreased in abundance in response to cold acclimation in the Christie background. Comparatively, 287 and 120 spots respectively increased and decreased in abundance in response to cold acclimation in the Endure background.

**Fig. 3:**
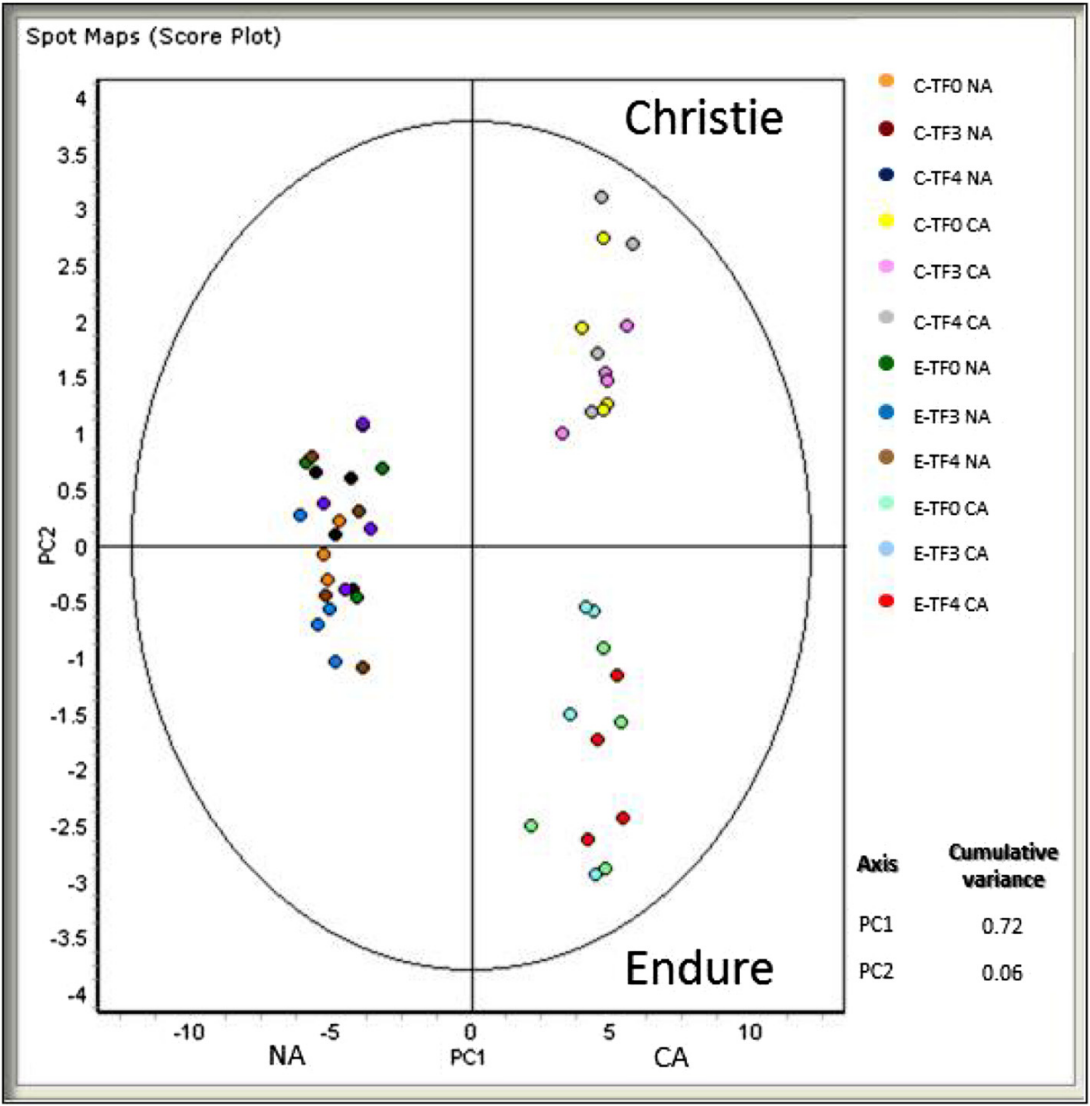
Principal component analysis of red clover proteome in response to cold acclimation and recurrent selection. Principal component analysis of proteome composition in crowns of red clover was based on two-dimensional difference gel electrophoresis (2D-DIGE). Analysis was performed with non-acclimated (NA) and cold-acclimated (CA) plants of the cultivar Christie (C) and the cultivar Endure (E) and populations TF3 and TF4 obtained after three and four cycles of recurrent selection in each initial genetic background (TF0). Variation in the proteome in relation to the cold acclimation, genetic background and recurrent selection is illustrated along the first two principal components (PC). Cumulative proportion of variations explained by PC1 and PC2 is indicated.

Analysis of principal components (PCA) was performed on the 627 proteins data set to assess how variations in the red clover proteome are related to the cold acclimation treatment and to the response to genetic selection in the two cultivars (Fig. 3). The first principal component (PC1) revealed that the largest source of variability (accounting for 72 % of the total variation) in the protein abundance was attributable to the cold acclimation treatment. Interestingly, the second principal component (PC2) accounting for 6 % of the total variation shows that populations from the two cultivars which were clustered in the non-acclimated state became highly differentiated after cold acclimation (Fig. 3).

### Common changes in protein abundance in response to cold acclimation

Spots with cold-acclimated to non-acclimated abundance ratios ≥ |1.50| in the three populations of the two genetic backgrounds (6 populations total) and with significant differences (*t*-test, *P* < 0.05) in at least five of the six populations, were used as robust criteria for the identification of proteins commonly cold-induced in red clover. Using that approach, 108 proteins among the 408 spots with homologies in databases increased (Additional file 3: Table S4a) while 33 proteins decreased in abundance in cold-acclimated compared to non-acclimated crowns in all populations of both cultivars (Additional file 4: Table S4b). These proteins are considered as being commonly cold-regulated and were subsequently grouped into up- and down-regulated proteins.

#### Up-regulated proteins

**Fig. 4:**
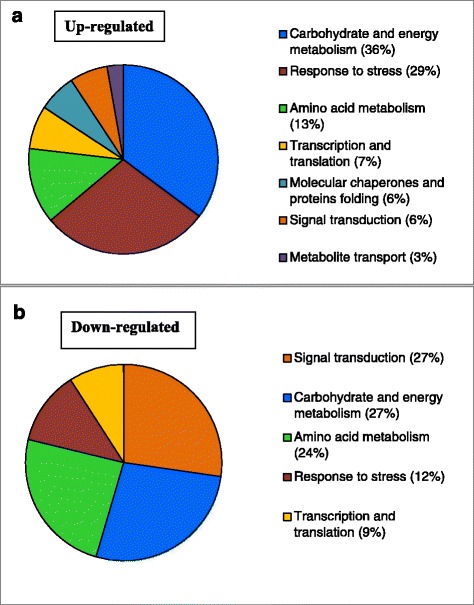
Functional classification of cold-responsive proteins commonly up-regulated (**a**) and down-regulated (**b**) in red clovers

The 108 protein spots that increased significantly in abundance in cold-acclimated crowns in red clover populations (Additional file 3: Table S4a) had homologies to forty-six (46) sequences in protein databases (Table 2). These sequences were assigned to seven functional groups (Fig. 4a) including response to stress (29 %), carbohydrate and energy metabolism (36 %), amino acid metabolism (13 %), signal transduction (6 %), molecular chaperones and protein folding (6 %), transcription and translation (7 %) and metabolite transport (3 %) (Fig. 4a).

**Table 2:** DIGE-spots with homology with sequences in databases that were up-regulated in response to cold acclimation

Spot no.	Homology	Functional category	CA/NA ratio
2039	Cold responsive protein TRVSP	Response to stress	29.54
1648	KS-dehydrin	Response to stress	20.81
2051	Cold acclimation-specific protein	Response to stress	11.03
1776	Actin-depolymerizing factor 2-like	Signal transduction	9.43
1601	Peptidyl-prolyl cis-trans isomerase 1-like	Molecular chaperones and protein folding	5.92
1587	Phospholipid hydroperoxide glutathione peroxidase	Response to stress (ROS scavenging)	5.14
547	Enolase-like	Carbohydrate and energy metabolism	5.02
93	Elongation factor 2-like	Transcription and translation	4.37
353	2,3-bisphosphoglycerate-independent phosphoglycerate mutase	Carbohydrate and energy metabolism	4.26
2046	Tyrosine phosphatase	Signal transduction	4.10
802	Monodehydroascorbate reductase	Response to stress (ROS scavenging)	4.07
142	Methionine synthase	Amino acid metabolism	4.05
1711	Ubiquitin-conjugating enzyme	Molecular chaperones and protein folding	3.92
318	NADP-dependent malic enzyme	Carbohydrate and energy metabolism	3.57
810	3-hydroxyisobutyryl-CoA hydrolase-like	Signal transduction (Cold stress signalling)	3.45
1365	Glutathione S-transferase	Response to stress (ROS scavenging)	3.38
1286	Mitochondrial outer membrane protein porin	Metabolite transport	3.32
887	Glyceraldehyde-3-phosphate dehydrogenase	Carbohydrate and energy metabolism	3.32
1280	Outer plastidial membrane protein porin-like	Metabolite transport	3.29
278	Stress-induced-phosphoprotein	Signal transduction	3.17
760	Transaldolase-like	Carbohydrate and energy metabolism	3.12
598	Elongation factor 1-gamma	Transcription and translation	3.08
378	Protein disulfide isomerase-like protein precursor	Molecular chaperones and protein folding	2.98
1510	Universal stress protein A-like protein	Response to stress	2.95
664	Abscisic acid stress ripening protein	Signal transduction	2.89
1369	Ferritin-2	Response to stress (ROS scavenging)	2.88
458	Adenosylhomocysteinase-like	Amino acid metabolism	2.87
1095	Isoflavone reductase related protein	Response to stress (ROS scavenging)	2.73
619	Elongation factor 1-alpha-like	Transcription and translation	2.65
66	Aconitate hydratase	Carbohydrate and energy metabolism	2.55
814	Fructose-bisphosphate aldolase	Carbohydrate and energy metabolism	2.40
2048	Malate dehydrogenase	Carbohydrate and energy metabolism	2.35
694	Isocitrate dehydrogenase [NADP]	Carbohydrate and energy metabolism	2.30
283	Heat shock protein	Molecular chaperones and proteins folding	2.20
842	Adenosine kinase	Carbohydrate and energy metabolism	2.18
1267	Proteasome subunit alpha type	Molecular chaperones and protein folding	2.00
527	Phosphopyruvate hydratase	Carbohydrate and energy metabolism	1.89
41	2-oxoglutarate dehydrogenase E1 subunit-like	Carbohydrate and energy metabolism	1.89
792	Glutamine synthetase	Amino acid metabolism	1.86
939	Quinone oxidoreductase-like protein	Response to stress (ROS scavenging)	1.81
772	Aspartate aminotransferase	Amino acid metabolism	1.81
1430	Chalcone isomerase 2	Response to stress (ROS scavenging)	1.80
769	Formate dehydrogenase 1	Carbohydrate and energy metabolism	1.78
667	Citrate synthase-like protein	Carbohydrate and energy metabolism	1.74
971	Fructokinase-2-like	Carbohydrate and energy metabolism	1.73
580	6-phosphogluconate dehydrogenase, decarboxylating-like	Carbohydrate and energy metabolism	1.70

For each spot, the name of the homologous sequence, the functional category and the average CA/NA abundance ratio across the six populations are listed. Spots with cold acclimated (CA) to non-acclimated (NA) abundance ratio ≥ 1.5 and with t-test, *P* < 0.05 in at least five populations are presented. In cases when homologous sequences were detected at different positions on the gels, the spot with the highest CA/NA ratio is presented. The exhaustive list of polypeptides up-regulated in response to cold acclimation is provided in Additional file 3: Table S4a

The group of proteins showing the largest increase in abundance in response to cold (up to 30 fold) were proteins related to the stress response category. These proteins also represented 29 % of the cold-induced proteins in all populations. This group is characterized by the presence of several variants of vegetative storage proteins from *Trifolium repens* (TRVSP) (up to 30 fold increase), KS-dehydrins (range of 9 to 21-fold increase) and a cold-acclimation specific (CAS) protein (11-fold increase) (Table 2 and Additional file 3: Table S4a). It also includes proteins involved in the scavenging of reactive oxygen species (ROS) and flavonoid metabolisms.

Proteins involved in metabolism of carbohydrates and energy production represent the most important functional category (36 %) with an average 2- to 5-fold increase in abundance in response to cold acclimation. Twenty-two proteins are involved in glycolysis, nine in the tricarboxylic acid (TCA) cycle, three in pentose phosphate pathway and an adenosine kinase which is involved in cellular energy homeostasis. Four non-photosynthetic nicotinamide adenine dinucleotide phosphate (NADP)-malic enzymes involved in the production of reductive power were also included in this category.

Among proteins up-regulated between four and two fold in response to cold acclimation, 14 proteins are involved in amino acid metabolism: ten were identified as methionine synthase, two as adenosylhomocysteinase, an asparate aminotransferase and a glutamine synthetase. Seven spots identified as signal transduction-related proteins including a tyrosine phosphatase (4-fold), and a stress-induced phosphoprotein (2 to 3-fold) increased in abundance in cold acclimated populations of both cultivars.

Peptides involved in protein translation or with homologies with chaperones and protein folding including a heat shock protein (HSP), an ubiquitin-conjugating enzyme 2, a proteasome subunit and elongation factors, increased in abundance in response to cold acclimation.

Three proteins involved in metabolite transport including two mitochondrial outer membrane protein porin and an outer plastidial membrane protein porin-like increased under cold acclimation.

#### Down-regulated proteins

Thirty three protein spots decreased significantly in abundance in cold-acclimated crowns of all the six red clover populations (Additional file 4: Table S4b). These spots were aligned with twelve homologous sequences (Table 3) that could be grouped into five functional categories (Fig. 4b): signal transduction (27 %), carbohydrate and energy metabolism (27 %), amino acid metabolism (24 %), stress response-related proteins (12 %) and transcription and translation (9 %).

**Table 3:** DIGE-spots with homology with sequences in databases that were down-regulated in response to cold acclimation

Spot no.	Homology	Functional category	CA/NA ratio
130	Sucrose synthase	Carbohydrate and energy metabolism	−3.51
545	Tubulin alpha	Signal transduction	−3.40
1660	Glycine-rich RNA-binding protein	Response to stress	−3.14
1325	6-phosphogluconolactonase	Carbohydrate and energy metabolism	−2.65
169	Methionine synthase	Amino acid metabolism	−2.57
1182	14-3-3-like protein	Signal transduction	−2.48
471	ATP synthase subunit beta	Carbohydrate and energy metabolism	−2.46
1694	Pathogenesis-related protein 10a	Response to stress (Defense, PR protein)	−2.39
605	Elongation factor 1-alpha-like	Transcription and translation	−2.39
700	Actin-101-like	Signal transduction	−2.35
1310	Thaumatin-like protein	Response to stress (Defense, PR protein)	−2.23
2037	Isoflavone reductase family protein	Response to stress	−1.84

For each spot, the name of the homologous sequence, the functional category and the average CA/NA abundance ratio across the six populations are listed. Spots with cold acclimated (CA) to non-acclimated (NA) abundance ratio ≤− 1.5 and with t-test, *P* < 0.05 in at least five populations are presented. In cases when homologous sequences were detected at different positions on the gels, the spot with the lowest CA/NA ratio is presented. The exhaustive list of polypeptides up-regulated in response to cold acclimation is provided in Additional file 4: Table S4b

Nine spots identified as actin-101 (two isoforms), tubulin (five isoforms) and 14-3-3-like protein (two isoforms), involved in signal transduction, decreased in abundance in all populations of both cultivars in response to cold acclimation. Nine protein spots associated with carbohydrate and energy metabolism were down-regulated in all populations of both cultivars in response to cold acclimation including five isoforms of sucrose synthase, three isoforms of adenosine triphosphate (ATP) synthase subunit beta. Eight proteins spots identified as methionine synthase decreased in abundance (2 to 3 fold) in all populations of both cultivars in response to cold acclimation. Four proteins associated with stress response including a thaumatin-like protein, a pathogenesis related protein, a glycine-rich ribonucleic acid (RNA)-binding protein 3 and an isoflavone reductase decreased in response to cold acclimation. Three isoforms of an elongation factor 1-alpha decreased in abundance in all populations of both cultivars in response to cold acclimation.

### Cultivar-specific changes in red clover proteome

Several identified proteins exhibited differential changes in abundance in response to cold acclimation between the two genetic backgrounds (Additional file 1: Table S2 and Additional file 2: Table S3). This includes a eukaryotic translation initiation factor 5A (spot #1560) which significantly increased in abundance in all three populations of Christie in response to cold acclimation while it remained unchanged in Endure (Table 4). Interestingly, the abundance of this eukaryotic translation initiation factor 5A increased with the number of selection cycles in the Christie background. A peroxiredoxin (spot #1683) decreased (3 fold) in all populations of cultivar Christie under cold acclimation while it remained unchanged in Endure background (Table 4).

**Table 4:** Proteins that varied in abundance in response to cold acclimation in a single genetic background

Cultivar	Cold regulation	Spot no.	Homology	Function	Average CA/NA protein ratio			
					TF0	TF3	TF4	Average
Christie	Increased	1560	Eukaryotic translation initiation factor 5A-like	Transcription and translation	4.8	8.12	10.01	7.64
	Decreased	1683	Peroxiredoxin	Response to stress (ROS scavenging)	−3.17	−3.79	−2.34	−3.1
Endure	Increased	74	Aconitate hydratase	Carbohydrate and energy metabolism (TCA cycle)	2.25	2.23	3.39	2.62
	Increased	18	Nuclease domain-containing protein	Transcription and translation (RNA stability)	2.21	2.44	2.76	2.47
	Increased	595	Elongation factor 1-gamma	Transcription and translation	2.52	2.14	2.52	2.39
	Increased	92	Elongation factor 2-like	Transcription and translation	2.32	2.06	2.19	2.19
	Increased	64	Puromycin-sensitive aminopeptidase-like	Protein metabolism	2.14	1.88	1.7	1.91
	Increased	586	Elongation factor 1-gamma	Transcription and translation	2.06	1.82	1.59	1.82
	Increased	594	Elongation factor 1-gamma-like	Transcription and translation	1.77	1.81	1.6	1.73
	Increased	68	Peptidase M1 family aminopeptidase N	Protein metabolism	1.85	1.57	1.52	1.65
	Decreased	1732	Disease resistance response protein 1	Response to stress (defense, PR protein)	−3.31	−1.81	−2.99	−2.70

Cold-acclimated (CA) to non-acclimated (NA) protein abundance that statistically differed (Student's *t*-test, *P* < 0.01) and with a CA/NA ratio ≥ |1.5| in all populations of the genetic background are presented. TF0 = original genetic background; TF3 and TF4 = populations produced after three and four cycles of selection for freezing tolerance, respectively

Nine proteins that were not cold-responsive in Christie populations varied significantly in response to cold acclimation in all populations of the cultivar Endure (Table 4). It includes, eight proteins up-regulated in response to cold acclimation including one aconitate hydratase (spot #74), four elongation factors involved in protein translation (spots #595, #92, #586 and #594), and three proteins involved in deoxyribonucleic acid (DNA) and protein quality control: a nuclease domain-containing protein (spot #18), a peptidase M1 family aminopeptidase (spot #68) and a puromycin-sensitive aminopeptidase (spot #64). The increase of abundance of the aconitate hydratase in acclimated crowns of the cultivar Endure after four selection cycles is noteworthy. A disease resistance response protein (spot #1732) down-regulated in cold-regulated populations of the cultivar Endure remained stable in populations of the Christie background (Table 4).

### Proteomic changes in response to recurrent selection for freezing tolerance

The abundance of only a few cold-regulated proteins significantly varied in response to recurrent selection for freezing tolerance (Table 5). For instance, levels of peptides with homologies with a 26S protease regulatory subunit 6A (spot # 665) and an actin 101-like protein (spot # 669) were 1.7- to 1.9-fold lower in cold-acclimated crowns of C-TF3 and C-TF4 than in C- TF0. The accumulation of a triosephosphate isomerase (spot # 1328) in non-acclimated crowns was 2.0- and 1.9-fold higher in C-TF4 than in C- TF0 and CTF3 respectively. Conversely, levels of a eukaryotic translation initiation factor 5A4 (spot # 1609) and a glyceraldehyde-3-phosphate dehydrogenase (spot # 909) were 1.5- to 1.7-fold higher in cold-acclimated crowns of C-TF3 and C-TF4 than in C-TF0 (Table 5).

**Table 5:** Proteins with significant variations in abundance in response to recurrent selection for freezing tolerance

				Ratio						Statistical significance of variation with a two-way ANOVA		
				Non-acclimated			Cold-acclimated					
Cultivar	Spot no.	Homology	Function	TF3/TF0	TF4/TF0	TF4/TF3	TF3/TF0	TF4/TF0	TF4/TF3	Cold acclimation	Populations	Cold acclimation * populations
Christie	665	26S protease regulatory subunit 6A homolog	Protein metabolism	−1.5	−1.3	1.1	**−1.9**	**−1.7**	1.1	***	**	NS
	669	Actin-101-like	Signal transduction	−1.3	−1.5	−1.2	**−1.9**	**−1.9**	1.0	***	**	NS
	1328	Triosephosphate isomerase	Carbohydrate and energy metabolism	1.1	**2.0**	**1.9**	1.2	1.5	1.3	***	***	NS
	1609	Eukaryotic translation initiation factor 5A4	Protein metabolism (Transcription and translation)	**2.5**	**2.4**	−1.0	1.5	**1.6**	1.1	***	**	NS
	909	Glyceraldehyde-3-phosphate dehydrogenase	Carbohydrate and energy metabolism	−1.3	1.4	**1.8**	**1.6**	**1.7**	1.0	NS	**	**
Endure	1648	KS-dehydrin	Response to stress	1.4	1.2	−1.2	**3.4**	**3.8**	1.1	***	**	NS
	1032	Annexin 1	Signal transduction	−1.3	−1.4	−1.1	1.3	**1.5**	1.2	***	NS	**
	1684	Glycine-rich RNA-binding protein	Signal transduction	1.8	1.3	−1.4	**2.0**	−1.4	**−2.7**	***	**	NS
	1656	Pathogenesis-related protein 10a	Response to stress (Defense, PR protein)	1.5	1.3	−1.1	**1.7**	1.3	−1.4	***	**	NS
	1694	Pathogenesis-related protein 10a	Response to stress (Defense, PR protein)	1.2	1.1	−1.2	**1.9**	1.3	−1.5	***	**	NS
	1722	Pathogenesis-related protein 10a	Response to stress (Defense, PR protein)	1.4	1.1	−1.3	**1.6**	1.3	−1.3	***	**	NS

Significant ratios (≥ |1,5|, *P* < 0.05 in Student's *t*-test) under cold-acclimated and non-acclimated conditions are indicated in bold. TF0 = original genetic background; TF3 and TF4 = populations produced after three and four cycles of selection for freezing tolerance, respectively. The statistical significance of variation with a two-way ANOVA is also shown. NS, **, ***: not significant, significant difference at *P* < 0.01 and *P* < 0.001 in a two-way analysis of variance (ANOVA), respectively

In the Endure genetic background, two up-regulated proteins including a KS-dehydrin (spot # 1648) and an annexin 1 (spots # 1032), and a down-regulated glycine-rich RNA-binding protein (spot # 1684) differentially accumulated in response to recurrent selection (Table 5). The abundance of a KS-dehydrin in cold-acclimated crowns of Endure increased with selection cycles. Three isoforms of pathogenesis-related protein 10a (spots # 1656, 1694 and 1722) and a glycine-rich RNA-binding protein (spot # 1684) were down-regulated in cold-acclimated crowns of Endure but were 1.6 to 2-fold more abundant in E-TF3 than in E-TF0 (Table 5).

## Discussion

### Recurrent selection improves freezing tolerance of red clover

Our results clearly demonstrated the capacity for substantial cold acclimation in red clover with a LT_50_ ranging from an average of −3 °C in non-acclimated plants down to −14 °C in plants overwintering under natural conditions in January. They also revealed that recurrent selection performed indoor is an effective approach to improve the freezing tolerance of red clover since the LT_50_ was improved by up to 3 °C after four cycles of selection (TF4) in the two genetic backgrounds. This is comparable to the 3 to 5 °C increase in freezing tolerance in alfalfa [4] and ryegrass [5] achieved with that selection protocol [2]. The positive impact of selection on red clover capacity to survive exposure to low subfreezing temperatures was further confirmed by the superior regrowth after freezing in cold-acclimated plants of populations TF3 and TF4 as compared to plants of TF0. It is expected that additional cycles of selection will further improve the freezing tolerance of red clover since results obtained with alfalfa, another open-pollinated forage species, showed that LT_50_ was still increasing after six cycles of selection [8].

### Cold acclimation causes extensive changes in the red clover proteome

The elucidation of molecular mechanisms underlying the acquisition of cold tolerance is required to deepen our understanding of the genetic bases of that complex trait and to facilitate the development of new breeding strategies to increase freezing tolerance. In the current study one of our objectives was to document the extent and nature of the changes in protein composition in crowns of red clover after long term (four months) acclimation to low temperature in fall and winter. Winter survival of red clover relies on subterranean crowns where meristems that provide root and shoot regrowth in the spring are located. Very robust criteria were used to identify cold-induced proteins including a protein abundance ratio higher than 1.50 for acclimated over non-acclimated crowns, and a significant variation in abundance between the two conditions in at least five of the six populations under study. Using that approach 36 % (146) of the 408 proteins with homologies with protein sequences in databases were deemed as commonly varying in abundance in response to cold acclimation. These cold-regulated proteins were assigned to functional categories based on their putative roles and potential involvement in the cold acclimation process.

For several cold-regulated proteins we noted the presence of isoforms (up to 23). This could be due to a number of factors including the fact that a single gene can give rise to different transcripts as a result of post-transcriptional (alternative RNA splicing, RNA editing, etc.) and post-translational modifications (phosphorylation, acetylation, methylation, ubiquitination, myristoylation, etc.) [12]. Isoforms can also arise from complex gene families with highly homologous variant sequences [9]. These isoforms can result in functionally and structurally distinct proteins with unique responses to the environment [13]. This may be the case in our study as different isoforms of some proteins (e.g. methionine synthase, isoflavone reductase) exhibited contrasting responses to cold acclimation. This suggests that specific isoforms may be involved in the cold acclimation process and/or the acquisition of superior levels of freezing tolerance.

#### Stress-response proteins

Sequences homologous to cold responsive vegetative storage proteins (VSP), KS-dehydrins and a cold acclimation-specific (CAS) protein were among the proteins that showed the highest increase in abundance (up to 30-fold) in response to cold acclimation. Report by Degand et al. [14] of similar accumulations of VSPs, dehydrins and CAS 15 in cold-acclimated roots of chicory (*Cichorium intybus*) is noteworthy and suggests their broad implication in the adaptation of perennial species to harsh winter conditions. The accumulation of VSPs has been linked with the overwintering potential and the vigor of spring regrowth in alfalfa [15] and white clover (*Trifolium repens* L.) [16]. There are several reports in the literature of a close relationship between the accumulation of dehydrins and the acquisition of freezing tolerance [17, 18]. In alfalfa, the presence of specific dehydrin variants has been linked to superior freezing tolerance levels [19, 20]. Multiple roles have been attributed to dehydrins, including cryoprotection, membrane and protein stabilization in freeze-desiccated cells and ROS scavenging [21]. Cold-induced KS-dehydrins possess a K segment which has been shown to be essential for cryoprotection [22, 23]. K_n_S-type dehydrins were also shown to have the ability to scavenge hydroxyl radicals and to bind metals [24]. The accumulation of *CAS* transcripts and proteins has been previously related to freezing tolerance in alfalfa [25–27]. The simultaneous increase in abundance of these three stress-proteins seems to be of major importance for the acquisition of freezing tolerance in red clover.

Stress-induced accumulation of ROS can cause damages to cellular components such as proteins, lipids, and nucleic acids [28]. Several ROS scavenging enzymes were up-regulated in cold-acclimated crowns of red clover including monodehydroascorbate reductase and glutathione reductase which are involved in the ascorbate-glutathione cycle, an important antioxidant system [29]. These two enzymes were previously reported to be up-regulated under cold acclimation in jack pine (*Pinus banksiana* Lamb.) [30], chicory [14], and winter wheat (*Triticum aestivum*) [31]. Cold acclimation also induced the accumulation of a hydroperoxide glutathione peroxidase (PHGPx) that has the ability to reduce highly reactive stess-induced phospholipid peroxides that accumulate in membranes [32–34]. We also observed the accumulation of universal stress-protein A and of ferritin-2, both reported to be involved in defence against ROS [35, 36]. Taken together, these observations confirm previous reports that proteome changes related to ROS protection is an important component of the cold acclimation process [11].

Cold acclimation induced the accumulation of enzymes involved in the biosynthesis of flavonoids. The levels of many flavonoid synthesis genes were recently correlated with freezing tolerance in 54 accessions of *Arabidopsis thaliana* and it was concluded that flavonoid metabolisms play an important role in freezing tolerance in that species [37]. One of the reported role of flavonol glucosides was to depress the freezing point of xylem parenchyma cells and to exhibit high anti-ice nucleation activity in katsura tree (*Cercidiphyllum japonicum*) [38]. Unexpectedly some proteins that have been associated with freezing tolerance were down-regulated in cold-acclimated crowns of red clover in January. This includes a thaumatin-like protein, a pathogenesis-related protein 10a [39], a glycine-rich RNA binding protein [40] and an isoflavone reductase [41]. As previously discussed, these down-regulated polypeptides may be functionally distinct isoforms. Other explanations of these discrepancies could be that their role in enhancement of freezing tolerance could wear off with the length of the cold acclimation period which was of 4 months (16 weeks) in our experiment as compared to 1 day to 7 weeks in other reports, or could be linked to the differences in experimental conditions and plant species.

#### Central metabolism and energy production

Cold acclimation is an active process associated with *de novo* biosynthesis of several stress-related metabolites that relies on the activation of several metabolic pathways and the production of high levels of energy [12]. A large proportion (35 %) of proteins that were identified in crowns of red clover was allocated to the “Carbohydrate and energy metabolism” functional category. Energy supply in heterotrophic cells relies mainly on glycolysis, pentose phosphate pathway (PPP), TCA cycle, and mitochondrial electron transport. We noted a decline in the levels of a 6-phosphogluconolactonase, a key enzyme of the oxidative phase of the PPP, along with an increased abundance of several enzymes of the TCA cycle and of NADP-malic enzymes in acclimated crowns. This is indicative of an increased contribution of the TCA cycle over the oxidative PPP, and of malate metabolism to provide the energy in overwintering red clover crowns. However, the increase of 6-phosphogluconate dehydrogenase and transaldolase in cold-acclimated crowns suggest that the reductive phase of PPP may still contribute to the protection against ROS as previously reported [42–44].

We observed the down-regulation of three proteins homologous to ATP synthase beta subunit in response to cold acclimation. Lower levels of this protein may facilitate the uncoupling of electron transport from ATP synthesis and as a result, a reduction of ROS formation caused by electron leakage [45]. We also detected the down-regulation of several sucrose synthase isoforms in cold-acclimated crowns of red clover. Decrease of sucrose synthase activity would promote the accumulation of sucrose, an important cryoprotectant [46], essential for the acquisition of freezing tolerance in alfalfa [47].

#### Amino acids metabolism

Cold acclimation induced the accumulation of ten isoforms of methionine synthase, an enzyme that catalyzes the final step in the regeneration of methionine from homocysteine. Methionine could then be used as an intermediate in the biosynthesis of polyamines and ethylene [48, 49]. The variation in the abundance of isoforms of methione synthase has recently been reported in a proteomic analysis of cold acclimated potato (*Solanum tuberosum*) [50] and velvet bentgrass (*Agrostis canina*) [9]. The protective effects of polyamines has been reported for drought, chilling and freezing stress in many plant species [51], while ethylene was shown to affect freezing tolerance [52] and to elicit antifreeze activity in non-acclimated winter ryegrass (*Lolium multiflorum*) [53]. Finally, we observed the up-regulation of isocitrate dehydrogenase and 2-oxoglutarate dehydrogenase in cold acclimated crowns. These enzymes are involved in the biosynthesis of proline which is a non-enzymatic ROS scavenger and a compatible solute [28].

#### Protein folding and molecular chaperones

We detected the cold-induced accumulation of proteins involved in the regulation of protein folding. Since freezing temperatures are associated with an enhanced risk of protein misfolding, the observed up-regulation of HSPs and molecular chaperones in red clover could protect against freeze-induced damage by maintaining proper protein conformation [54, 55]. For instance, HSPs exert a strong cryoprotective effect through its action on membrane protection, in the refolding of denatured proteins, and in preventing protein aggregation [56]. Peptidyl-prolyl cis-trans isomerase 1-like and protein disulfide isomerase-like protein have also demonstrated chaperone activity [57] and were reported to be up-regulated under cold acclimation of various plant species [55, 58]. We observed a cold-induced increased in the abundance of an ubiquitin-conjugating enzyme and in three isoforms of proteasome subunit alpha type that are both involved in the degradation of ubiquitin-tagged proteins [59]. These findings suggest that cold acclimation of red clover relies on the ubiquitin/proteasome-mediated proteolysis, a post-transcriptional process that has been shown to play crucial roles in cold signaling and cold tolerance by modulating the selective degradation of regulatory proteins [60, 61].

#### Metabolite transport

Ion transport and compartmentation is of great importance in cellular homeostasis during stress [62]. Freezing temperatures reduce the membrane fluidity that modulates ion channel activity and could disrupt cellular homeostasis [63]. The outer mitochondrial membrane is generally permeable to specific metabolites due to the presence of porins, which are proteins forming large channels and allowing diffusion of small molecules to maintain cell homeostasis [64]. The up-regulation of these ion channel-related proteins played an important role in in the acquisition of freezing tolerance in barley (*Hordeum vulgare*) [65]. In this perspective, the cold-induced accumulation of two mitochondrial outer membrane porins and of an outer plastidial membrane protein porin could be involved in the maintenance of membrane permeability in cold-acclimated crowns of red clover.

#### Signal transduction-related proteins

The up-regulation of actin-depolymerizing factor 2-like and the down-regulation of actin-101-like proteins and of alpha and beta tubulins in cold-acclimated red clover suggests a cytoskeleton reorganization which is prerequisite to the acquisition of cold-induced freezing tolerance [66]. Microtubules and actin filaments modulate the activity of Ca^2+^ channels following membrane rigidification [67] which is considered a primary cold-sensing response [68]. Abscisic acid (ABA) mediates ROS-signal transduction and is a key regulatory of multiple genes associated with freezing tolerance in plant [69]. The accumulation of an ABA stress ripening protein suggests that cold-induced freezing tolerance in red clover was at least partially related to an ABA-dependent regulatory pathway.

Protein phosphorylation and dephosphorylation are major stress signaling events that initiates the expression of cold-regulated genes [70–72]. Our observation of cold-induced accumulation of phosphoproteins, tyrosine phosphatase and adenosine kinase in red clover could be associated to that role.

### Differences between the two genetic backgrounds Christie and Endure

PCA analysis of the variability in protein abundance shows that populations from the two cultivars were clustered together in the non-acclimated state and became highly differentiated after cold acclimation. This result clearly shows that each cultivar has specific cold responses involving the accumulation of a subset of proteins. This underscores the fact that the acquisition of freezing tolerance within a species can be achieved through common and distinct protein components. This may explain frequent reports of contrasting proteomic and genetic responses using forward and reverse gene experiments. For instance, a protein involved in the elongation step during mRNAs translation (translation initiation factor 5A-like) was found to be up-regulated in the cultivar Christie only. Conversely, three isoforms of elongation factor-1 gamma and one elongation factor-2 were down-regulated only in the cultivar Endure. The progressive increase in the abundance of the translation initiation factor 5A-like in response to selection is noteworthy and warrants further investigation of that protein and/or encoding gene as a potential marker for selection. This protein has been linked with an increase in ROS scavenging capacity and an increase tolerance to stress in transgenic *Arabidopsis* and *Nicotiana benthamiana* [73, 74].

Cold acclimation induced the accumulation of isoforms of elongation factor 1-gamma and elongation factor 2-like proteins only in cultivar Endure. In addition to its function in the synthesis of the polypeptide chain, eukaryotic elongation factor 1-gamma has been involved in post-translational regulation of glutathione-mediated detoxification mechanisms in plants under oxidative stress [75]. Earlier study showed that elongation factor 2 is required for cold-induced transcription and the development of freezing tolerance in *Arabidopsis thaliana* [76]. Accordingly, our results suggest that these proteins contribute to the improvement of freezing tolerance in Endure. These observations concur with reports that the regulation of mRNA translation is a key process of plant adaptation to stress [77]. It also suggests that mRNAs translation is differentially regulated in the two red clover genetic backgrounds.

A peptidase M1 family aminopeptidase N, a predicted puromycin-sensitive aminopeptidase-like isoform X3 and a nuclease domain-containing protein were also cold-induced specifically in Endure. Both aminopeptidases are involved in the selective degradation of misfolded, damaged and specific regulatory proteins [78, 79]. Their cold-induction might help avoid accumulation of oligopeptides at toxic levels in cytosol and to prevent oligopeptides interference with important protein-protein interactions [80, 81]. Our results also showed the progressive induction of an aconitate hydratase in response to selection only in the cultivar Endure. Aconitate hydratase (aconitase) is a key regulatory enzyme of the TCA cycle that plays a role in the antioxidant defense system by preventing excessive accumulation of ROS [82]. Espevig et al. [9] associated the cold-induction of aconitate hydratase to the improvement of freezing tolerance in velvet bentgrass. Few proteins were differentially down-regulated between the two genetic backgrounds. For instance, a peroxiredoxin decreased in intensity only in the cultivar Christie while a PR-1 decreased only in Endure. Gaudet et al. [83] observed a decline in transcript levels of PR proteins in winter wheat acclimated at −3 °C.

### Proteomic changes associated with recurrent selection

Only a few proteins were associated with the response to selection. Interestingly, these proteins differed between cultivars Christie and Endure. In Endure, an isoform of KS-dehydrin increased gradually with increasing the number of selection cycles (3.4 fold in E-TF3 and 3.8-fold in E-TF4 over E-TF0). Many studies reported that dehydrins isoforms could be used as reliable markers of plant freezing tolerance in various species including alfalfa [19], barley, wheat [31] and white clovers [18]. We observed a similar increase in an annexin 1 homolog in response to selection in Endure. Annexins have been shown to be involved in ROS regulation and in stress signalling [84]. Other proteins increased in response to three cycles of selection and then remained at the same level in response to a fourth cycle. In Christie, two proteins involved in carbohydrate and energy metabolism increased gradually in response to selection: a glyceraldehyde-3-phosphate dehydrogenase (GAPDH) and a triose phosphate isomerase (TPI). Enhanced glycolysis is an adaptive response of plants to generate energy to cope with environmental stress [85]. The upregulation of GAPDH and TPI suggests that enzymes of the glycolytic pathway were under selection pressure for freezing tolerance at least in one of the two genetic backgrounds of red clover.

The fact that only few proteins are associated with recurrent selection that led to a 2 to 3 °C increase in LT_50_ shows the importance of these proteins for the acquisition of freezing tolerance in red clover. This may also imply that selection leads to small increment of several proteins instead of a few major changes when it comes to a complex trait such as cold tolerance.

## Conclusion

Our results clearly demonstrate the capacity for substantial cold acclimation in red clover. The two functional groups of proteins that increased the most in response to cold acclimation were “stress-response proteins” including vegetative storage proteins, dehydrins, and ROS scavenging enzymes, and “carbohydrate and energy metabolism” proteins. The improvement of freezing tolerance by recurrent selection was associated with differential accumulation of a small number of cold-regulated proteins that differed between the two genetic backgrounds. The fact that a limited number of proteins varied significantly in response to recurrent selection suggest that they may play an important role in the determination of the level of freezing tolerance or may also reflect the fact that response to recurrent selection rely on the small increment of several proteins.

## Methods

### Plant material

Experimentations on plants were performed indoor under controlled environment and comply with national and international guidelines.

### Selection for freezing tolerance

Recurrent phenotypic selection for freezing tolerance was performed using the procedure described in Bertrand et al. (2014) [2] and was independently applied to red clover cultivars Endure [86] and Christie [87]. To avoid genetic drift and to maintain genetic diversity, a broad base of 1500 genotypes was used at each cycle of selection. Plants were started from seeds in 164 mL-volume pots (Ray Leach Cone-tainers, SC Super Cell, low density) placed in RL 98 trays (Stuewe and sons Inc., Tangent, OR, USA). After 5 weeks of growth in a growth chamber set to 22/17 °C (day/night) temperatures, 600-800 μmol photons m^−2^ s^−1^ photosynthetic photons flux density (PPFD) during a 16 h-photoperiod, plants were transferred to a constant temperature of (2 °C) and 150 μmol photons m^−2^ s^−1^ PPFD under a short photoperiod (8 h) for two weeks followed by two weeks at −2 °C in the dark. After that acclimation period, plants were exposed to a sub-freezing temperature near the LT_50_ to eliminate freezing sensitive plants. After three rounds of growth-acclimation-freezing stress, the 100 most vigorously re-growing genotypes were intercrossed in a greenhouse using bumblebees to generate a new population putatively more tolerant to freezing (TF). Using that approach, four cycles of selection were performed within each genetic background. The original backgrounds Christie and Endure are respectively identified as C-TF0 and E-TF0 in the manuscript. Populations obtained from the recurrent selection process were sequentially identified as C-TF1 to C-TF4 and E-TF1 to E-TF4.

### Freezing tolerance and biomass of regrowth

Freezing tolerance of populations C-TF0 and E-TF0 and populations C-TF3, C-TF4, E-TF3 and E-TF4 was assessed before (October 2010) and after cold acclimation (January and February 2011), using a procedure described in [4]. Briefly, in September 2010, fifteen plants were seeded in 14-cm pots and allowed to establish under controlled environment under the following conditions: photoperiod, 16 h; 600-800 μmol photons m^−2^ s^−1^ PPFD; day-time temperature, 22 °C; night-time temperature, 17 °C. After 1 wk, plants were thinned to 10 per pot and, after 5 wk of growth, pots were transferred to an unheated greenhouse located at a site near Québec City, Canada (latitude, 46°47'15"; longitude 71°12'00"; altitude, ≈ 45 m asl) for their acclimation to natural hardening conditions. When the air temperature inside the greenhouse remained permanently below freezing, plants were covered with a layer of Astrofoil reflection insulator (Innovative Energy Inc., St-Louis, MO) to simulate snow cover. Air temperature outside and inside the greenhouse and soil temperature in pots were monitored at 30-min intervals and recorded from the end of October 2010 to February 2011 using stand-alone dataloggers (Model-RD-TEMP-XT; Omega Engineering Inc.). Freezing tests were performed in a programmable walk-in freezer following a 24 h equilibration period at −2 °C. Temperatures were lowered by 2 °C during a 30-min period followed by a 90-min plateau at each test temperature. Plants were tested between −2 and −6 °C in October and between −10 to −20 °C in January and February. At the end of each temperature plateau, five pots (50 plants) were withdrawn from the freezer and thawed at 2 °C for 24 h. Plants were then transferred to initial growth conditions and, after three weeks, survival counts in each pot were determined. Lethal temperature for 50 % of the plants (LT_50_) of the initial backgrounds (TF0) and populations TF3 and TF4 was then computed using the SAS Probit procedure [88]. Biomass of regrowth after freezing was also recorded as an additional indicator of plant damage after freezing. Biomass was measured after exposure to −13.0 °C, a temperature close to the LT_50_ of the initial genetic backgrounds C-TF0 and E-TF0. Biomass in each pot was assessed by collecting the top growth after a 3 wk-regrowth period. Samples were dried at 55 °C in an air-forced oven for 48 h to determine the regrowth dry biomass and divided by the number of plants per pot.

### Proteomic analysis

#### Tissue sampling

Samples were collected from non-acclimated plants in October 2010 and from cold-acclimated plants in January 2011. In January, pots were thawed overnight at 4 °C prior to samples collection. At each sampling date, roots from five pots (10 plants pot^−1^) of each population were washed free of soil under a stream of cold water. Crowns with buds (5-cm transition zone between shoots and roots) were separated from the roots and cut into small segments (4-5 mm) with a chopper to obtain homogeneous samples. For each pot, 1 g of fresh weight (FW) of the pooled sample from 10 plants was freeze-dried for 48 h. Lyophilized samples were ground with a Mixer Mill (MM 310, Retsch GmbH & Co. KG, Haan, Germany) and stored at room temperature until proteomic analyses.

#### Total soluble proteins extraction

Total soluble proteins were extracted from lyophilized material using a trichloroacetic acid (TCA)/acetone precipitation method followed by a sodium dodecyl sulfate (SDS) and phenol extraction method as follows: 300 mg of ground lyophilized crowns was suspended in 1 mL of 20 % (w/v) ice-cold TCA in acetone with 0.1 % (w/v) dithiothreitol (DTT) and kept for 60 min at −20 °C to allow protein precipitation. Samples were centrifuged at 10000 x *g* for 5 min at 4 °C. The pellets were washed with 1.5 mL ice-cold acetone and centrifuged again at 10000 x *g* for 5 min at 4 °C. The washing step was repeated once and pellets were subsequently dried under vacuum.

Pellets were re-suspended in 0.6 mL UltraPure™ Buffer-Saturated Phenol (Invitrogen) and 0.6 mL SDS buffer [30 % (w/v) sucrose, 2 % (w/v) SDS, 0.1 M Tris-HCl, pH 8.0, 5 % (v/v) 2-mercaptoethanol]. Samples were shaken using an Eppendorf Thermomixer at 1400 rpm for 20 min at 20 °C and subsequently centrifuged at 10000 x *g* for 5 min at 20 °C to allow the separation of the phenolic and the SDS-buffer phases.

For each sample, 300 μL from the phenolic phase were collected in 2 mL microtubes. Proteins were precipitated by addition of 1.5 mL ice-cold 0.1 M ammonium acetate in methanol and kept for 30 min at −20 °C. Samples were centrifuged at 10000 x *g* for 5 min at 4 °C and the pellets were washed twice with ice-cold 0.1 M ammonium acetate in methanol. Samples were washed two more times in ice-cold acetone/water (80/20 (v/v)) and re-suspended in labelling buffer [7 M urea, 2 M thiourea, 30 mM Tris, 2 % (w/v) 3-[(3-Chlolamidopropyl) dimethylammonio]-1-propanesulfonate (CHAPS)]. Samples were shaken on an Eppendorf Thermomixer at 700 rpm for 60 min at 20 °C, centrifuged for 5 min at 14000 x *g* to remove insoluble material. Prior to quantification, the pH of the samples was adjusted to 8.5 using 1 M NaOH. The quantification of solubilized proteins was carried out with a Bradford protein assay using bovine serum albumin (2 mg mL^−1^) as standard.

#### Protein analysis

##### Two-dimensional difference gel electrophoresis (2D-DIGE)

For each sample, 50 μg of proteins was labelled with 400 pmole of cyanine dyes Cy3 or Cy5 LUMIPROBE LLC 25 nmol (Interchim®). Two samples were randomly assigned to each gel (Additional file 5: Table S1). A pooled sample combining equal amounts of proteins from each of the 48 samples was labelled with 400 pmol of Cy2 dye and used as internal standard for gel normalization. Labelling of samples and the internal standard was carried out in the dark at 4 °C. After 30 min of incubation, the reaction was quenched by adding 1 μL of 10 mM lysine followed by further 10 min of incubation in the dark at 4 °C. On each gel, the Cy2-labelled internal standard was mixed with the Cy3- and Cy5-labelled protein extracts (50 μg of each) and the volume was adjusted to 150 μL with a rehydration buffer [7 M urea, 2 M thiourea, 0.5 % (w/v) CHAPS]. Samples were mixed with 3 μL of non-linear (NL) immobilized pH gradient (IPG) buffer (pH 3-10 NL) (GE Healthcare) and then loaded by cup loading onto 24 cm Immobiline™ DryStrip Gels (GE Healthcare) previously rehydrated overnight in 450 μL Destreak Rehydration Solution (GE Healthcare) mixed with 0.5 % non-linear IPG buffer (pH 3-10 NL) (GE Healthcare). The first dimension of the 2D gel electrophoresis involved the separation of proteins according to their isoelectric point (PI) by isoelectric focusing carried out at 20 °C in an Ettan IPGphor III system (GE Healthcare) following a 5-step program: (1) constant 150 V for 2 h, (2) 4 h linear gradient from 150 V to 1000 V, (3) constant 1000 V for 5 h, (4) 5 h linear gradient from 1000 V to 10000 V, (5) constant 10000 V voltage until reaching a total of around 90000 V-h. During IEF, the maximum current setting was 75 μA *per* strip.

Prior to the second-dimension electrophoresis by sodium dodecyl sulfate-polyacrylamide gel electrophoresis (SDS-PAGE), IPG strips were equilibrated for 15 min in an equilibration buffer (Serva Electrophoresis GmbH, Heidelberg) complemented with 6 M Urea and 1 % w/v DTT. This was followed by an additional 15 min in the same equilibration buffer containing 2.5 % w/v iodoacetamide. Strips were subsequently loaded on 2D HPE™ Large Gels NF 12.5 % w/v acrylamide (Serva Electrophoresis GmbH). SDS-PAGE was carried out using an HPE™ Flatbed Tower System according to manufacturer’s instructions. After the front reached the bottom of the gel, the proteins were fixed in a solution containing 15 % v/v ethanol and 1 % m/v of citric acid for at least 2 h and rinsed with MilliQ water (Millipore Corporation). Gels were subsequently scanned using a Typhoon FLA 9500 scanner (GE Healthcare) and quantitative analysis was carried out using the DeCyder software (v7.0, GE Healthcare).

##### Spot excision, digestion and identification of proteins

Reproducible spots present in at least 75 % of the gels were excised with an Ettan Spot Picker robot (GE Healthcare). Spots were collected in 96-well plates, washed and desalted with a 50 mM ammonium bicarbonate solution containing 50 % (v/v) methanol followed by 75 % (v/v) acetonitrile solution and dried at 37 °C. Proteins within each gel spot were digested for 6 h at 37 °C in 8 μL of trypsin Gold (5 ng/μL trypsin in 20 mM ammonium bicarbonate) (Promega, Madison, WI, USA). The resulting peptides were dissolved in 50 % (v/v) acetonitrile containing 0.1 % (v/v) trifluoroacetic acid (TFA), dried at 53 °C and spotted on matrix-assisted laser desorption/ionization (MALDI) plates. Then, 0.7 μL of α-cyano-4-hydroxycinnamic acid [7 mg mL^−1^ acetonitrile 50 % (v/v) containing TFA 0.1 % (v/v)] was added to each extracted peptide mixture. Digestion and spotting were done using the Tecan freedom EVO200 (Tecan, Männedorf, CH). For mass spectroscopy (MS) analysis, MALDI peptide mass spectra were acquired using the AB Sciex 5800 time-of-flight (TOF)/TOF (AB Sciex, Redwood City, CA). For subsequent MS/MS analysis, 10 most intense peaks, excluding known contaminants, were selected and fragmented.

All spectra, MS and MS/MS, were submitted for database-dependent identification using the NCBI non-redundant protein sequence database (http://www.ncbi.nlm.nih.gov) downloaded on June 6^th^, 2014 (40,910,947 sequences) reduced to the green plants Taxonomy (1,717,798 sequences) using an in-house MASCOT server (Matrix Science, www.matrixscience.com, London, U.K.). A secondary search was also carried out against an EST fabaceae database downloaded from the NCBI server on December 17^th^, 2013 (19,932,450 sequences). The parameters used for these searches were mass tolerance MS 100 ppm, mass tolerance MS/MS 0.5 Da, fixed modifications cysteine carbamidomethylation, and variable modifications methionine oxidation, simple and double oxidation of tryptophan, tryptophan to kynurenine. Proteins were considered as identified when at least two peptides passed the MASCOT-calculated 0.05 threshold score of 40. However when a high-scoring peptide (>2 times the 0.05 threshold) was matched and a protein *p*-value of < 1 × 10e-5 was obtained, the protein was also retained. Subsequently the MASCOT-based results were manually validated. The list of matched peptides was compared to the MS spectrum in order to reduce ambiguities caused by non-identified proteins co-migrating with resolved peptides. When high-intensity peaks were not matched to the identified protein, the corresponding MS/MS spectra were researched and/or the sequence of the peptide was determined de novo. The eventual matching of peaks in the MS spectrum to a second protein excluded these spots from biological interpretation. The matching of a peptide containing one of the oxidized forms of tryptophan was likewise verified by the presence of peaks corresponding to the same peptide with the other oxidation products of this residue (Trp; Trp +4 Da = kynurenine, Trp +16 Da = oxidized Trp; Trp +32 Da = N -formylkynurenine). This likewise resulted in the delineation of several signal and transit peptides and the discovery of different molecular forms of the same protein in some of the spots. MS/MS spectra resulting in a score around the MASCOT threshold (*P* < 0.05) of 40 were validated by using the following criteria: high intensity peaks should be matched and well-known sequence-dependent characteristics should be present. These easy-to-recognize spectral features include the presence of a peak corresponding to the C-terminal arginine and the presence of the neutral loss of 64 Da from peptides containing oxidized methionine. Furthermore, the impact that specific residues (most notably proline and aspartic acid) have on the intensity of fragment peaks must be respected. In case high quality spectra did not result in significant identification, manual *de novo* sequence analysis was applied and/or extra peaks were fragmented to confirm near-to-threshold identifications. The mass spectrometry proteomics data have been deposited to the ProteomeXchange Consortium via the PRIDE [88] partner repository with the dataset identifier PXD003689.

### Statistical analyses

#### Freezing tolerance (LT_50_) and biomass of regrowth

The experiment on freezing tolerance and biomass regrowth was conducted according to a completely randomized block design with four blocks. The experimental unit was a pot containing 10 plants. Statistical analyses were performed using the general linear models (GLM) procedure of the SAS software package [89]. Analysis of variance and multiple comparisons (Protected least significant difference, LSD) were used to compare populations within each genetic background. Statistical significance was set at *P* <0.05. LT_50_ values were calculated using the Probit procedure of SAS, and the difference between treatments was evaluated using a chi-square goodness of fit test as described in [4].

#### Proteomic data

Within each cultivar, changes in individual protein abundances were assessed by combination of Student’s -test and a two way-analysis of variance (ANOVA), with cold acclimation treatment as factor one and number of selection cycles as factor two. For each protein spot, significant changes (increase or decrease) in abundance levels were assessed using the threshold of significance *p* < 0.01 and average ratios (Cold-acclimated/Non-acclimated) of protein abundances ≥ |1.50|. The plant sample distribution in the multivariate space defined by the proteome composition was inferred by Principal Component Analysis (PCA).

## Availability of data and materials

All relevant data are within the paper and its Supporting Information files.

Proteome data were deposited in the ProteomeXchange database under the accession number PXD003689.

Plant material: Red clover Christie and Endure initial backgrounds are commercial cultivars. Recurrent selection is breeding material developed at the Quebec Research and Development Centre of Agriculture and Agri-Food Canada and is available upon request.

## Additional files

Additional file 1: Table S2. List of spots analyzed in the initial background Christie (C-TF0) and recurrently selected populations after three (C-TF3) and four cycles (C-TF4) and statistical analyses of the effects of cold acclimation, selection and their interactions on the relative abundance in crowns. (XLSX 218 kb) Additional file 2: Table S3. List of spots analyzed in the initial background Endure (E-TF0) and recurrently selected populations after three (E-TF3) and four cycles (E-TF4) and statistical analyses of the effects of cold acclimation, selection and their interactions on the relative abundance in crowns. (XLSX 221 kb) Additional file 3: Table S4a. List of DIGE-spots with homology with sequences in databases that are up-regulated in response to cold acclimation (ANOVA, *P* < 0.01). (XLSX 27 kb) Additional file 4: Table S4b. List of DIGE-spots with homology with sequences in databases that are down-regulated in response to cold acclimation (ANOVA, *P* < 0.01) (XLSX 18 kb) Additional file 5: Table S1. Use of the cyanine (Cy) dyes to label the different samples. (XLSX 12 kb)

## References

[1] Bosworth SC, Stringer WC. Cutting management of alfalfa, red clover and birdsfoot trefoil. www.forages.psu.edu/agfacts/agfact7.pdf (1990). Accessed 20 Jul 2015.

[2] Bertrand A, Castonguay Y, Bourassa J, Hincha DK, Zuther E (2014). A whole-plant screening test to identify genotypes with superior freezing tolerance. Plant Cold Acclimation: Methods and protocols, Methods in Molecular Biology.

[3] Castonguay Y, Dubé M-P, Cloutier J, Bertrand A, Michaud R, Laberge S (2013). Molecular physiology and breeding at the crossroads of cold hardiness improvement. Physiol. Plant..

[4] Castonguay Y, Michaud R, Nadeau P, Bertrand A (2009). An indoor screening method for improvement of freezing tolerance in alfalfa. Crop Sci..

[5] Iraba A, Castonguay Y, Bertrand A, Floyd DJ, Cloutier J, Belzile FJ (2013). Characterization of populations of turf-type perennial ryegrass recurrently selected for superior freezing tolerance. Crop Sci..

[6] Guy CL, Kaplan F, Kopka J, Selbig J, Hincha DK (2008). Metabolomics of temperature stress. Physiol. Plant..

[7] Thomashow MF (2010). Molecular basis of plant cold acclimation: insights gained from studying the CBF cold response pathway. Plant Physiol..

[8] Castonguay Y, Bertrand A, Michaud R, Laberge S (2011). Cold-induced biochemical and molecular changes in alfalfa populations selectively improved for freezing tolerance. Crop Sci..

[9] Espevig T, Xu C, Aamlid TS, DaCosta M, Huang B (2012). Proteomic responses during cold acclimation in association with freezing tolerance of velvet Bentgrass. J. Amer. Soc. Hort. Sci..

[10] Chen K, Renaut J, Sergeant K, We H, Arora R (2013). Proteomic changes associated with freeze-thaw injury and post-thaw recovery in onion (*Allium cepa* L.) scales. Plant Cell Environ.

[11] Chen J, Han G, Shang C, Li J, Zhang H, Liu F, Wang J, Liu H, Zhang Y (2015). Proteomic analyses reveal differences in cold acclimation mechanisms in freezing-tolerant and freezing-sensitive cultivars of alfalfa. Front Plant Sci..

[12] Kosová K, Vítámvás P, Urban MO, Klίma M, Roy A, Prášil IT (2015). Biological networks underlying abiotic stress tolerance in temperate crops- A proteomic perspective. Int. J. Mol. Sci..

[13] Rowley ER, Mockler TC, Shanker A, Venkateswarlu B (2011). Plant abiotic stress: insights from the genomics era. Abiotic Stress Response in Plants - Physiological, biochemical and genetic perspectives.

[14] Degand H, Faber AM, Dauchot N, Mingeot D, Watillon B, Cutsem PV, Morsomme P, Boutry M (2009). Proteomic analysis of chicory root identifies proteins typically involved in cold acclimation. Proteomics.

[15] Avice JC, Le Dily F, Goulas E, Noquet C, Meuriot F, Volenec JJ, Cunningham SM, Sors TG, Dhont C, Castonguay Y, Nadeau P, Bélanger G, Chalifour F–P, Ourry A (2003). Vegetative storage proteins in overwintering storage organs of forage legumes, roles and regulation. Can. J. Bot.

[16] Goulas E, Le Dily F, Ozouf J, Ourry A (2003). Effects of a cold treatment of the root system on white clover (*Trifolium repens* L.) morphogenesis and nitrogen reserve accumulation. J. Plant Physiol.

[17] Zhang X, Wang K, Ervin EH (2008). Bermudagrass freezing tolerance associated with abscisic acid metabolism and dehydrin expression during cold acclimation. J. Amer. Soc. Hortic. Sci..

[18] Vaseva II, Anders I, Feller U. Identification and expression of different dehydrin subclasses involved in the drought response of *Trifolium repens*. J Plant Physiol. 2014;171213–24. 10.1016/j.jplph.2013.07.013 24054754

[19] Rémus-Borel W, Castonguay Y, Cloutier J, Michaud R, Bertrand A, Desgagnés R, Laberge S (2010). Dehydrin variants associated with superior freezing tolerance in alfalfa (*Medicago sativa* L.). Theor. Appl. Genet..

[20] Dubé M-P, Castonguay Y, Cloutier J, Michaud J, Bertrand A (2013). Characterization of two novel cold-inducible K3 dehydrin genes from alfalfa (*Medicago sativa* spp. sativa L.). Theor. Appl. Genet.

[21] Hanin M, Brini F, Chantal E, Toda Y, Takeda S, Masmoudi K (2011). Plant dehydrins and stress tolerance versatile proteins for complex mechanisms. Plant Signal. Behav..

[22] Reyes JL, Campos F, Wei HUI, Arora R, Yang Y, Karlson DT, Covarrubias AA (2008). Functional dissection of hydrophilins during in vitro freeze protection. Plant Cell Environ.

[23] Yang W, Zhang L, Lv H, Li H, Zhang Y, Xu Y, Yu J (2015). The K-segments of wheat dehydrin WZY2 are essential for its protective functions under temperature stress. Front. Plant Sci..

[24] Rorat T (2006). Plant dehydrins-tissue location, structure and function. Cell. Mol. Biol. Lett..

[25] Monroy AF, Castonguay Y, Laberge S, Sarhan F, Vezina LP, Dhindsa RS (1993). A new cold-induced alfalfa gene is associated with enhanced hardening at subzero temperature. Plant Physiol..

[26] Pennycooke JC, Cheng HM, Stockinger EJ (2008). Comparative genomic sequence and expression analyses of *Medicago truncatula* and alfalfa subspecies falcata cold-acclimation-specific genes. Plant Physiol..

[27] Zhang LL, Zhao MG, Tian QY, Zhang WH (2011). Comparative studies on tolerance of *Medicago truncatula* and *Medicago falcata* to freezing. Planta.

[28] Gill SS, Tuteja N (2010). Reactive oxygen species and antioxidant machinery in abiotic stress tolerance in crop plants. Plant Physiol. Biochem..

[29] Das K, Roychoudhury A (2014). Reactive oxygen species (ROS) and response of antioxidants as ROS-scavengers during environmental stress in plants. Front. Environ. Sci..

[30] Zhao S, Blumwald E (1998). Changes in oxidation-reduction state and antioxidant enzymes in the roots of jack pine seedlings during cold acclimation. Physiol. Plant..

[31] Kosová K, Vítámvás P, Prášilová P, Prášil IT (2013). Accumulation of WCS120 and DHN5 proteins in differently frost-tolerant wheat and barley cultivars grown under a broad temperature scale. Biol. Plant..

[32] Yang XD, Dong CJ, Liu JY (2006). A plant mitochondrial phospholipid hydroperoxide glutathione peroxidase: its precise localization and higher enzymatic activity. Plant Mol. Biol..

[33] Faltin Z, Holland D, Velcheva M, Tsapovetsky M, Roeckel-Drevet P, Handa AK, Abu-Abied M, Friedman-Einat M, Eshdat Y, Perl A (2010). Glutathione peroxidase regulation of reactive oxygen species level is crucial for *in vitro* plant differentiation. Plant Cell Physiol..

[34] Jain P, Bhatla SC (2014). Signaling role of phospholipid hydroperoxide glutathione peroxidase (PHGPX) accompanying sensing of NaCl stress in etiolated sunflower seedling cotyledons. Plant Signal Behav.

[35] Nachin L, Nannmark U, Nystrom T (2005). Differential roles of the universal stress proteins of Escherichia coli in oxidative stress resistance, adhesion, and motility. J. Bact..

[36] Deák M, Horváth GV, Davletova S, Török K, Sass L, Vass I, Barna B, Király Z, Dudits D (1999). Plants ectopically expressing the iron-binding protein, ferritin, are tolerant to oxidative damage and pathogens. Nat. Biotechnol..

[37] Schulz E, Tohge T, Zuther E, Fernie AR, Hincha DK (2015). Natural variation in flavonol and anthocyanin metabolism during cold acclimation in *Arabidopsis thaliana* accessions. Plant Cell Environ..

[38] Kasuga J, Hashidoko Y, Nishioka A, Yoshiba M, Arakawa K, Fujikawa S (2008). Deep supercooling xylem parenchyma cells of katsura tree (*Cercidiphyllum japonicum*) contain flavonol glycosides exhibiting high anti-ice nucleation activity. Plant Cell Environ..

[39] Hon WC, Griffith M, Mlynarz A, Kwok YC, Yang DS (1995). Antifreeze proteins in winter rye are similar to pathogenesis-related proteins. Plant Physiol..

[40] Kim YO, Kim JS, Kang H (2005). Cold-inducible zinc finger-containing glycine-rich RNA-binding protein contributes to the enhancement of freezing tolerance in *Arabidopsis thaliana*. Plant J..

[41] Miura K, Furumoto T (2013). Cold signaling and cold response in plants. Int. J. Mol. Sci..

[42] Dennis DT, Blakeley SD. Carbohydrate metabolism. In: Buchanan B, Gruissem W, Jones RL, editors, Biochemistry and Molecular Biology of Plants. American Society of Plant Physiologists, Rockville, MD; 2000. p 630–75.

[43] Mittler R (2002). Oxidative stress, antioxidants and stress tolerance. Trends Plant Sci..

[44] Corpas FJ, Barros JB (2014). NADPH-generating dehydrogenases: their role in the mechanism of protection against nitro-oxidative stress induced by adverse environmental conditions. Front. Plant Sci..

[45] Fernie AR, Carrari F, Sweetlove LJ (2004). Respiratory metabolism: glycolysis, the TCA and mitochondrial electron transport. Curr. Opin. Plant Biol..

[46] Uemura M, Steponkus PL (2003). Modification of the intracellular sugar content alters the incidence of freeze-induced membrane lesions of protoplasts isolated from Arabidopsis thaliana leaves. Plant Cell Environ..

[47] Castonguay Y, Nadeau P (1998). Enzymatic control of soluble carbohydrate accumulation in cold-acclimated crowns of alfalfa. Crop Sci..

[48] Ravanel S, Gakiere B, Job D, Douce R (1998). The specific features of methionine biosynthesis and metabolism in plants. Proc. Natl. Acad. Sci. USA.

[49] Hesse H, Kreft O, Maimann S, Zeh M, Hoefgen R (2004). Current understanding of the regulation of methionine biosynthesis in plants. J. Exp. Bot..

[50] Renaut J, Planchon S, Oufir M, Hausman JF, Hoffmann NL, Evers D, Gusta L, Wisniewski M, Tanino K (2009). Identification of proteins from potato leaves submitted to chilling temperature. Plant Cold Hardiness: from the laboratory to the field.

[51] Calzadilla PI, Gazquez A, Maiale SJ, Ruiz OA, Bernardina MA. Polyamines as indicators and modulators of the abiotic stress in plants. In: Anjum NA, Gill SS, Gill R, editors. Plant adaptation to environmental change: Significance of amino acids and their derivatives. CABI, Wallingford, UK; 2014. p. 109–28.

[52] Zhang ZJ, Huang RF (2010). Enhanced tolerance to freezing in tobacco and tomato overexpressing transcription factor TERF2/LeERF2 is modulated by ethylene biosynthesis. Plant Mol. Biol..

[53] Yu XM, Griffith M, Wiseman SB (2001). Ethylene induces antifreeze activity in winter rye leaves. Plant Physiol..

[54] Wang WX, Vinocur B, Shoseyov O, Altman A (2004). Role of plant heat-shock proteins and molecular chaperones in the abiotic stress response. Trends Plant Sci..

[55] Feng R, Zhang L, Wang J, Luo J, Peng M, Qi J, Zhang Y (2015). Proteomic Analysis of Cold Stress Responses in Banana Leaves. J. Amer. Soc. Hort. Sci..

[56] Suzuki N, Mittler R (2006). Reactive oxygen species and temperature stresses: A delicate balance between signaling and destruction. Physiol. Plant..

[57] Boston RS, Viitanen PV, Vierling E (1996). Molecular chaperones and protein folding in plants. Plant. Mol. Biol..

[58] Vítámvás P, Prášil IT, Kosová K, Planchon S, Renaut J. Analysis of proteome and frost tolerance in chromosome 5A and 5B reciprocal substitution lines between two winter wheats during long-term cold acclimation. Proteomics. 2012;1268–85. 10.1002/pmic.201000779 22065556

[59] Stone S (2014). The role of ubiquitin and the 26S proteasome in plant abiotic stress signaling. Front Plant Sci..

[60] Chinnusamy V, Zhu J-K, Sunkar R (2010). Gene regulation during cold stress acclimation in plants. Methods Mol. Biol..

[61] Miura K, Ohta M (2010). SIZ1, a small ubiquitin-related modifier ligase, controls cold signaling through regulation of salicylic acid accumulation. J. Plant Physiol..

[62] Conde A, Chaves MM, Gerós H (2011). Membrane transport, sensing and signaling in plant adaptation to environmental stress. Plant Cell Physiol..

[63] Los DA, Murata N (1666). Membrane fluidity and its roles in the perception of environmental signals. BBA– Biomembranes..

[64] Schwarzländer M, Finkemeier I (2013). Mitochondrial energy and Redox signaling in plants. Antioxid. Redox Signal..

[65] Baldi P, Grossi M, Pecchioni N, Vale G, Cattivelli L (1999). High expression of a gene coding for a chloroplastic amino acid-selective channel protein is correlated to cold acclimation in cereals. Plant Mol. Biol..

[66] Ruelland E, Zachowski A (2010). How plants sense temperature. Environ. Exp. Bot..

[67] Wasteneys GO, Yang Z (2004). New views on the plant cytoskeleton. Plant Physiol.

[68] Örvar BL, Sangwan V, Omann F, Dhindsa RS (2000). Early steps in cold sensing by plant cells: the role of actin cytoskeleton and membrane fluidity. Plant J..

[69] Gusta LV, Trischuk R, Weiser CJ (2005). Plant cold acclimation: the role of abscisic acid. J. Plant Growth Regul..

[70] Sharma P, Sharma N, Deswal R (2005). The molecular biology of the low-temperature response in plants. Bioessays.

[71] Xiong L, Schumake KS, Zhu JK (2002). Cell signaling during cold, drought, and salt stress. Plant Cell.

[72] Meskiene I, Baudouin E, Schweighofer A, Liwosz A, Jonak C, Rodriguez PL, Jelinek H, Hirt H (2003). The stress-induced protein phosphatase 2C is a negative regulator of a mitogen-activated protein kinase. J. Biol. Chem..

[73] Xu J, Zhang B, Jiang C, Ming F (2011). RceIF5A, encoding an eukaryotic translation initiation factor 5A in *Rosa chinensis*, can enhance thermotolerance, oxidative and osmotic stress resistance of *Arabidopsis thaliana*. Plant Mol. Biol..

[74] Wang L, Xu C, Wang C, Wang Y (2012). Characterization of a eukaryotic translation initiation factor 5A homolog from *Tamarix androssowii* involved in plant abiotic stress tolerance. BMC Plant Biol..

[75] Jain M, Ghanashyam C, Bhattacharjee A (2010). Comprehensive expression analysis suggests overlapping and specific roles of rice glutathione-S-transferase genes during development and stress responses. BMC Genomics.

[76] Guo Y, Xiong L, Ishitani M, Zhu JK (2002). An *Arabidopsis* mutation in translation elongation factor 2 causes superinduction of CBF/DREB1 transcription factor genes but blocks the induction of their downstream targets under low temperatures. Proc. Natl. Acad. Sci. USA.

[77] Floris M, Hany M, Elodie L, Christophe R, Benoit M (2009). Post-transcriptional regulation of gene expression in plants during abiotic stress. Int. J. Mol. Sci..

[78] Sánchez-Moran E, Jones G, Franklin F, Santos J (2004). A puromycin sensitive aminopeptidase is essential for meiosis in Arabidopsis thaliana. Plant Cell.

[79] Peer WA (2011). The role of multifunctional M1 metallopepptidases in cell cycle progression. Ann. Bot..

[80] Saric T, Graef CI, Goldberg AL (2004). Pathway for degradation of peptis generated by proteasomes. A key role for thimet oligopeptidase and other metallopeptidases. J. Biol. Chem..

[81] Smalle J, Vierstra RD (2004). The ubiquitin 26 s proteasome proteolytic pathway. Annu. Rev. Plant Physiol. Plant Mol. Biol.

[82] Moeder W, del Pozo O, Navarre DA, Martin GB, Klessig DF (2007). Aconitase plays a role in regulating resistance to oxidative stress and cell death in *Arabidopsis* and *Nicotiana benthamiana*. Plant Mol. Biol..

[83] Gaudet DA, Wang Y, Frick M, Puchalski B, Penniket C, Ouellet T, Robert L, Singh J, Laroche A (2011). Low temperature induced defence gene expression in winter wheat in relation to resistance to snow moulds and other wheat diseases. Plant Sci..

[84] Clark G, Konopka-Postupolska D, Hennig J, Roux S (2010). Is annexin 1 a multifunctional protein during stress responses?. Plant Signal. Behav..

[85] Bertrand A, Prévost D, Bigras FJ, Castonguay Y (2007). Elevated atmospheric CO2 and strain of *Rhizobium* alter freezing tolerance and cold-induced molecular changes in alfalfa (*Medicago sativa*). Ann. Bot..

[86] Christie BR, Choo TM, Papadopoulos YA, Lewis J, Michaud R (1998). AC Endure red clover. Can. J. Plant Sci..

[87] Martin RA, Christie BR, Papadopoulos YA, Martin RC (1999). AC Christie red clover. Can. J. Plant Sci..

[88] Vizcaíno JA, Csordas A, del-Toro N, Dianes JA, Griss J, Lavidas I, Mayer G, Perez-Riverol Y, Reisinger F, Ternent T, Xu QW, Wang R, Hermjakob H (2016). 2016 update of the PRIDE database and related tools. Nucleic Acids Res.

[89] Institute SAS (1999). SAS/Stat user’s guide.

